# 

**Published:** 1909-06

**Authors:** 


### BOOKS RECEIVED.

The Practical Medicine Series. Ten volumes. Issued under the general editorial charge of Gustavus P. Head, M.D., Professor of Laryngology and Rhinology in the Chicago Post-Graduate Medical School. Vol. I, General Medicine, edited by Frank Billings, M.S., M.D. Head of the Medical Department and Dean of the Faculty of Rush Medical College. Series 1909. Chicago: The Year Book Publishers. (Price, $1.50; entire series, $10.00.)

The Practical Medicine Series. Ten volumes. Issued under the general editorial charge of Gustavus P. Head, M.D., Professor of Laryngology and Rhinology in the Chicago Post-Graduate Medical School. Vol. II, General Surgery, edited by John B. Murphy, A.M., M.D., LL.D., Professor of Surgery in the Northwestern University; Attending Surgeon and Chief of Staff, Mercy Hospital. Series 1909. Chicago: The Year Book Publishers. (Price, $2.00; entire series, $10.00.)

Writing the Short-Story. By J. Berg Esenwein, A.M., Lit. D. Editor of Lippincott's Monthly Magazine. Author of How to Attract and Hold an Audience. Cloth, 12mo, 448 pages. Price, $1.25. Published by Hinds, Noble & Eldredge, New York.

